# Natural Taste Modulators and Microbiome-Aware Nutritional Support for Immunotherapy-Associated Dysgeusia: A Translational Perspective for Precision Supportive Cancer Care

**DOI:** 10.3390/nu18142393

**Published:** 2026-07-22

**Authors:** Anna Fleischer

**Affiliations:** Department of Internal Medicine II/Psychosomatics, University Hospital Würzburg, 97080 Würzburg, Germany; fleischer_a@ukw.de

**Keywords:** dysgeusia, taste alteration, cancer immunotherapy, talquetamab, GPRC5D, nutrition, oral-gut microbiome, miraculin, miracle berry, supportive care, precision oncology

## Abstract

Dysgeusia is a clinically consequential, but still under-standardized, toxicity of cancer treatment. In the immunotherapy era, taste disturbances are increasingly relevant for patients receiving immune checkpoint inhibitors, chimeric antigen receptor (CAR) T-cell therapies and T-cell-redirecting bispecific antibodies, with G protein-coupled receptor family C group 5 member D (GPRC5D)-directed treatment in multiple myeloma representing a particularly instructive high-burden model. We performed a structured critical narrative review with evidence mapping. PubMed/MEDLINE was searched from database inception to June 2026, complemented by citation tracking in Google Scholar, ClinicalTrials.gov searches and guideline documents relevant to oncology nutrition, oral supportive care and cancer-related taste dysfunction. Search concepts covered cancer-related dysgeusia, immunotherapy-associated oral toxicity, GPRC5D/talquetamab-associated dysgeusia, oncology nutrition, oral–gut microbiome biology, natural taste modulators and miraculin-based interventions. Dysgeusia can reduce appetite, food enjoyment, dietary diversity and protein energy intake, thereby contributing to weight loss, malnutrition risk, distress, social withdrawal and, in severe cases, treatment modification or discontinuation. Available evidence is heterogeneous: general cancer-treatment-associated dysgeusia is supported by broader observational and interventional literature; immunotherapy-associated dysgeusia is less systematically characterized; and GPRC5D/talquetamab-associated dysgeusia represents the most clinically visible and target-specific immunotherapy-associated phenotype. Emerging pilot data suggest that dried miracle berry or miraculin-containing products may improve selected taste perception and nutritional parameters in cancer-related dysgeusia, but direct evidence in immunotherapy-associated dysgeusia is not yet established. We, therefore, propose a claim-disciplined precision supportive-care framework integrating systematic taste phenotyping, early nutritional risk assessment, oral health evaluation, microbiome-aware but hypothesis-generating endpoints, individualized flavor and texture adaptation, cautious use of natural taste modulators in selected patients and iterative monitoring of patient-centered outcomes. Future trials should test whether dysgeusia-focused nutritional and taste-modulating supportive care interventions can improve intake, quality of life and treatment persistence without compromising immunotherapy safety or efficacy.

## 1. Introduction

Cancer immunotherapy has transformed the treatment landscape of solid tumors and hematologic malignancies. Immune checkpoint inhibitors, chimeric antigen receptor (CAR) T-cell therapy and T-cell-redirecting bispecific antibodies can induce durable responses in selected patients, but they also create supportive-care needs that differ from classical cytotoxic therapy. Among these needs, taste and smell alterations remain under-standardized despite their consistent association with reduced food enjoyment, dietary restriction, malnutrition risk and impaired quality of life [1,2,3,4,5,6,7,8].

This problem is especially relevant for therapies that target antigens expressed on both malignant cells and physiologic tissues involved in oral function. Talquetamab, a GPRC5D × CD3 bispecific antibody, has shown robust activity in relapsed or refractory multiple myeloma [9,10]. At the same time, oral adverse events, xerostomia, dysgeusia, reduced appetite and weight loss have emerged as clinically meaningful toxicities of GPRC5D-directed therapy [11,12,13,14]. The biological plausibility is supported by GPRC5D expression in hard-keratinized structures and by preclinical work establishing GPRC5D as an immunotherapeutic target in multiple myeloma [15,16]. Across treatment modalities, the evidentiary landscape differs substantially. Taste and smell alterations after chemotherapy and radiotherapy have been described for many years and are supported by systematic reviews, meta-analyses and supportive-care literature, whereas immunotherapy-associated dysgeusia remains less standardized and more unevenly reported [17]. Within immunotherapy, GPRC5D-directed therapy is currently the most instructive model because dysgeusia, xerostomia, appetite loss and weight loss occur frequently and appear biologically linked to target expression in keratinized tissues. By contrast, evidence for taste dysfunction after immune checkpoint inhibitors and CAR T-cell therapy is more indirect and often embedded within broader oral toxicity, nutrition or quality-of-life reporting. This distinction is important because supportive care algorithms derived from chemotherapy or radiotherapy populations cannot be automatically transferred to bispecific antibody-treated or cellular-therapy-treated patients.

For this reason, the present review separates four evidence streams whenever possible: general cancer-related dysgeusia, malnourished oncology populations, immunotherapy-associated dysgeusia and GPRC5D/talquetamab-associated dysgeusia. This structure is intended to prevent overgeneralization and to clarify where recommendations are evidence-based, where they represent cautious extrapolation and where they remain hypothesis-generating.

Nutrition is the clinical bridge between sensory toxicity and meaningful outcomes. International cancer nutrition and cachexia guidance emphasizes early screening for nutritional risk, individualized counseling, protein energy adequacy, symptom-oriented nutrition impact management and multidisciplinary care [18,19,20,21,22]. However, standard algorithms are not yet fully adapted to patients whose dominant barrier to eating is not nausea, vomiting or mechanical swallowing impairment but altered flavor perception, disgust, loss of food reward and food-specific aversions during immunotherapy.

Natural products occupy an appealing but delicate position in this field. Natural compounds have been reviewed as modulators of antitumor immunity, tumor immune microenvironment biology, immune checkpoints, immunogenic cell death and intracellular immune signaling pathways [23,24,25,26,27,28]. However, clinical translation must avoid overstatement. For patients receiving immunotherapy, the most clinically actionable use of selected natural products may be as carefully selected, food-based, symptom-targeted supportive interventions that help preserve intake, microbiome resilience, quality of life and treatment persistence, while potential anticancer immunomodulatory effects should remain hypothesis-generating unless supported by clinical trials.

This review, therefore, develops a precision supportive care framework for immunotherapy-associated dysgeusia. It integrates nutritional assessment, oral–gut microbiome biology and natural taste modulators while preserving strict boundaries between supportive care hypotheses and anticancer claims. The aim is to provide a clinically useful and research-ready roadmap for a highly relevant but underdeveloped area of oncology care.

## 2. Methods: Scope and Structured Literature Approach

This article is a critical narrative review and translational perspective with structured evidence mapping. It was designed to identify clinically actionable concepts, safety boundaries and research priorities rather than to estimate pooled effect sizes. Because the literature spans several partially disconnected fields—cancer-related dysgeusia, immunotherapy-associated oral toxicity, oncology nutrition, oral and gut microbiome biology, natural products and miraculin-based taste modulation—a narrative format was selected. However, to improve transparency and reduce selective citation, the literature identification process followed a structured search logic.

PubMed/MEDLINE was searched from database inception through 16 June 2026 and updated on 16 July 2026. Four focal PubMed search streams were used for the structured evidence map: (1) cancer-related dysgeusia or taste disorders (92 records); (2) talquetamab/GPRC5D-related taste, xerostomia, oral toxicity or weight loss (33 records); (3) miraculin/miracle berry in cancer-related taste disorders or malnutrition (19 records); and (4) oral or gut microbiome studies linking cancer immunotherapy with diet, nutrition, antibiotic exposure or treatment response (219 records). The complete Boolean search strings are provided in Supplementary Table S1. The four searches yielded 363 records and 359 unique records after de-duplication. Complementary citation tracking in Google Scholar, a ClinicalTrials.gov search for ongoing or registered miraculin and dysgeusia studies, and targeted searches of relevant guideline and society documents were performed for oncology nutrition, cachexia, oral complications and supportive care.

The single author screened the titles and abstracts of all 359 unique PubMed records. Potentially eligible full texts and relevant sources identified by citation tracking were then assessed against prespecified conceptual eligibility criteria. Eligible sources were peer-reviewed clinical studies, observational studies, systematic or scoping reviews, highly relevant narrative reviews, mechanistic studies, guideline documents and trial protocols addressing at least one of the following domains: cancer-related dysgeusia; immunotherapy-associated taste or oral toxicity; GPRC5D/talquetamab-associated dysgeusia; nutritional consequences of taste dysfunction; natural taste modulators; miraculin or miracle berry interventions; oral or gut microbiome changes during cancer treatment; or microbiome-related immunotherapy outcomes. Sources were excluded if they were unrelated to cancer; did not address taste, nutrition, oral toxicity, microbiome biology or natural products; were not available in English; duplicated a more complete report without adding relevant information; or focused primarily on unsubstantiated anticancer supplement claims without supportive care relevance. The final narrative synthesis comprised 56 cited sources, including publications and guidelines added through citation tracking. Because this was a structured critical narrative review rather than a systematic review, no formal dual-reviewer screening, PRISMA flow diagram or risk-of-bias assessment was undertaken.

Evidence was narratively synthesized by both directness and methodological strength. Directness was categorized as: (1) direct evidence for GPRC5D/talquetamab-associated dysgeusia; (2) direct evidence for immunotherapy-associated dysgeusia or oral toxicity; (3) indirect evidence from chemotherapy, radiotherapy, head-and-neck cancer or general oncology nutrition; and (4) hypothesis-generating evidence from microbiome or natural product immunology studies. Methodological strength was appraised using prespecified criteria: population and outcome relevance, prospective versus retrospective design, presence of a comparator, sample size and attrition, use of validated or objective outcome measures, consistency across studies, and independence of datasets. In the evidence tables, ‘high’ or ‘moderate’ denotes comparatively robust and clinically relevant evidence; ‘low’ denotes small or methodologically limited clinical studies; and ‘very low’ denotes very small pilots, selected cohorts, secondary analyses or exploratory mechanistic findings. These labels are a narrative evidence appraisal and not a formal GRADE certainty rating. No meta-analysis was performed; conclusions are therefore framed as a translational supportive-care roadmap rather than clinical practice guidelines.

### Added Value Compared with Previous Reviews

Several recent reviews have addressed overlapping but distinct areas, including taste and smell alterations in cancer patients, nutritional management of oncological symptoms, oral microbiome alterations after cancer treatment, diet–microbiome–immunotherapy interactions and microbiome-targeted strategies to improve immunotherapy outcomes [3,8,29,30,31,32]. The present review differs from these publications in four ways. First, it focuses specifically on dysgeusia as a nutrition impact toxicity in the immunotherapy era rather than as a general cancer-treatment symptom. Second, it treats GPRC5D/talquetamab-associated dysgeusia as a model toxicity in which target biology, oral symptoms, nutritional burden and treatment persistence converge. Third, it distinguishes supportive use of natural taste modulators from unproven anticancer natural product claims. Fourth, it proposes trial-ready endpoints that connect sensory phenotype, nutritional outcomes, oral-gut microbiome biology, patient-reported outcomes and treatment adherence. The intended contribution is therefore not another general review of cancer-related taste alteration or microbiome-immunotherapy interactions, but a translational framework for precision supportive care in immunotherapy-associated dysgeusia.

Table 1 summarizes the relative evidence context across treatment settings. The purpose is to show why GPRC5D-directed treatment represents a particularly informative model for immunotherapy-associated dysgeusia while acknowledging that much of the broader supportive-care evidence still derives from chemotherapy, radiotherapy and general oncology populations.

## 3. Immunotherapy-Associated Dysgeusia as a Supportive Care Blind Spot

Taste alteration is common across oncology, but the field has historically focused more on mucositis, nausea, vomiting, diarrhea and cachexia than on the sensory mechanisms that make eating aversive. This is problematic because dysgeusia is not merely a symptom label. It is a pathway from treatment toxicity to reduced intake, dietary narrowing, lower protein and energy consumption, weight loss, distress and poorer quality of life [1,2,3,4,33,34]. Qualitative work further indicates that eating problems during oncology treatment affect social participation, identity and emotional well-being, not only nutrient intake [3,4].

For clinical interpretation, four evidence streams should be separated. First, general cancer-related dysgeusia provides the broadest symptom and supportive care background. Second, malnourished oncology populations provide evidence for nutritional risk, dietary counseling and selected taste modulating interventions, but are not necessarily immunotherapy-specific. Third, immunotherapy-associated dysgeusia is clinically plausible but remains less consistently captured across immune checkpoint inhibitor and CAR T-cell studies. Fourth, GPRC5D/talquetamab-associated dysgeusia currently provides the clearest high-burden immunotherapy model, because taste disturbance, dry mouth, appetite loss and weight loss are repeatedly reported as part of a target-associated oral toxicity cluster. Throughout this review, recommendations are therefore labeled as direct, indirect or hypothesis-generating according to their evidence source.

Terminology requires strict separation. Dysgeusia denotes a qualitative distortion of taste; hypogeusia denotes reduced taste sensitivity; ageusia denotes complete loss of taste; and altered flavor may reflect gustatory, olfactory or retronasal dysfunction. Xerostomia is the subjective sensation of dry mouth and is distinct from objectively measured hyposalivation. Dysphagia, stomatitis or oral mucositis, reduced appetite and composite oral symptom outcomes are separate clinical entities and should not be used as proxies for dysgeusia. Where source studies grouped ageusia, hypogeusia or unspecified taste disorder under an adverse event category labeled ‘dysgeusia,’ this review reproduces that grouping but states it explicitly in the study-level summary.

Immunotherapy-associated dysgeusia is heterogeneous. It may occur through immune-mediated oral inflammation, altered salivary composition, epithelial injury, neural dysfunction, microbiome disruption, infections, concomitant drugs or target-specific effects. In GPRC5D-directed therapy, the biological plausibility is particularly strong because the target is associated with keratinized structures and because talquetamab cohorts consistently report dysgeusia, xerostomia, appetite loss and weight loss [11,12,13,14,15,16].

The treatment context magnifies the burden. Patients receiving bispecific antibodies or cellular therapies are often heavily pretreated, immunocompromised, fatigued and nutritionally vulnerable. A symptom that might be tolerable in a short chemotherapy course may become unacceptable when it persists during continuous immunotherapy. Dysgeusia can also be socially isolating: meals may become stressful, family members may struggle to understand the complaint and patients may feel guilty when they cannot eat food prepared for them [13]. This psychosocial dimension is clinically important and should be assessed rather than dismissed.

Study-level talquetamab data are summarized in Table 2. The estimates should not be pooled: trial adverse-event reporting, retrospective CTCAE grading, cross-sectional psychophysical testing and symptom-selected qualitative cohorts answer different questions and use different denominators.

## 4. Pathophysiological Rationale: Taste Buds, Saliva, Oral Barrier and Microbiome

Taste perception is an integrated system involving taste receptor cells, epithelial renewal, saliva, oral microbial ecology, olfaction, trigeminal stimulation, central processing and emotional context. Oncology treatments can disrupt several components simultaneously. Taste bud turnover can be impaired by inflammation or epithelial injury; saliva can change in volume or composition; mucosal barrier dysfunction can expose nerve endings and alter oral ecology; and smell dysfunction can be misperceived as taste loss because flavor perception depends strongly on retronasal olfaction [35,36].

The oral microbiome is increasingly recognized as a dynamic ecosystem affected by cancer treatment. Chemotherapy, targeted therapy, immunosuppression and cellular therapies can shift oral microbial communities, favor opportunistic infection and alter inflammatory tone [29,37]. In dysgeusia, oral dysbiosis may be both cause and consequence: local inflammation and altered saliva may influence taste perception, while avoidance of specific foods, reduced oral intake and mucosal care practices may further reshape the oral ecosystem. Importantly, this pathway should be interpreted with caution. Direct causal evidence linking specific oral microbiome signatures to immunotherapy-associated dysgeusia is currently limited. The oral microbiome is therefore best considered a biologically plausible and clinically relevant exploratory domain rather than an established therapeutic target for dysgeusia management. In contrast, xerostomia, mucosal inflammation, candidiasis, dental disease, medication exposures and reduced intake are clinically observable contributors that should be assessed and treated directly.

The gut microbiome adds a systemic layer. Multiple studies have associated gut microbial composition with response to immune checkpoint blockade, while antibiotic exposure and low microbial diversity have been linked to worse outcomes in several settings [30,38,39,40,41,42,43]. Diet is one of the few modifiable inputs to the gut microbiome. High-fiber dietary patterns have been associated with favorable immunotherapy outcomes in melanoma, whereas indiscriminate probiotic supplementation may not be beneficial and should not be treated as a generic supportive care shortcut [31,32]. These gut microbiome data are highly relevant for immunotherapy biology, but they do not demonstrate that microbiome modulation improves dysgeusia. Most clinical evidence links microbial diversity, dietary fiber or antibiotic exposure to immune checkpoint inhibitor response, particularly in melanoma, rather than to taste function or bispecific antibody-associated oral toxicity. In this review, gut microbiome endpoints are, therefore, proposed as exploratory translational measures in future dysgeusia trials, not as validated clinical biomarkers for current dysgeusia management.

## 5. Nutritional Consequences and Clinical Assessment

Dysgeusia becomes clinically important when it changes eating behavior. Patients may avoid meat because it tastes metallic or bitter, reject water or coffee, overuse sweet foods because savory foods are intolerable, avoid social meals, or rely on low-protein comfort foods. These changes can reduce energy intake, protein adequacy, dietary diversity, micronutrient density and food-related quality of life. In heavily treated patients, even moderate weight loss can be clinically meaningful if it occurs alongside sarcopenia, fatigue, infections or treatment interruptions [12,44,45,46].

The clinical assessment should, therefore, move beyond asking whether taste is altered.

The key question is: Which foods have become impossible, which foods remain acceptable and what nutritional consequences follow? This requires a dysgeusia-oriented assessment that integrates objective testing, patient-reported symptom burden, oral health, olfaction, nutritional risk and patient priorities.

The following assessment framework combines established guideline-consistent elements, such as nutritional risk screening and oral complication assessment, with dysgeusia-specific expert interpretation. As summarized in Table 3, it separates the taste phenotype from smell/flavor, oral, nutritional, treatment context and psychosocial domains. It should be understood as a pragmatic supportive care workflow rather than a validated dysgeusia-specific guideline.

Validated tools can be combined pragmatically. Taste Strips provide objective gustatory assessment [36], EORTC QLQ-C30 captures appetite loss and global health status [47], PG-SGA [48] or NRS-2002 [49] can screen nutritional risk and short distress screens can detect emotional burden [50]. Digital tools may further support repeated monitoring, individualized food trials and patient-generated data. A clinically useful workflow should be lightweight enough for routine oncology visits but sensitive enough to trigger early dietitian, oral medicine or psycho-oncology involvement.

## 6. Natural Taste Modulators: Miraculin as a Leading Candidate

Miraculin is a taste-modifying glycoprotein from Synsepalum dulcificum, commonly known as miracle berry or miracle fruit. Under acidic conditions, miraculin changes taste perception by making sour foods taste sweet and can alter perceived intensities of other taste qualities [51]. This effect is particularly relevant for cancer dysgeusia because many patients find that acidic, cold or strongly flavored foods remain more tolerable than bland foods, while sweet foods or meat may become aversive. Miraculin does not correct the underlying cause of dysgeusia; rather, it may create a window in which certain foods become more acceptable.

Clinical evidence remains preliminary and should be interpreted as hypothesis-generating. Two independent interventional cohorts have been published. An eight-patient randomized crossover pilot suggested improved food palatability during miracle fruit exposure [52]. The CLINMIR program subsequently evaluated dried miracle berry in malnourished patients with cancer-related taste disorders using a randomized, triple-blind, placebo-controlled pilot design and companion oral microbiome, intestinal microbiome and biomarker analyses [51,53,54,55,56]. In the parent CLINMIR trial, 31 participants were randomized and 21 completed the three-month intervention. The standard-dose group showed improved electrogustometric taste acuity over time and higher salty-taste perception than placebo, together with signals in energy coverage, fat-free mass and selected quality-of-life outcomes [53]. The companion analyses identified exploratory oral and intestinal microbial and biomarker changes, but did not establish causality or clinical mediation [54,55,56]. Thus, miraculin is a plausible candidate for translational study because it has a defined sensory mechanism and early clinical feasibility data; however, it should not be presented as an established treatment for immunotherapy-associated dysgeusia. A practical implication is that miraculin should be evaluated as a sensory-supportive tool, not as a stand-alone nutritional therapy. Its effect depends on acidic food vehicles and individual sensory phenotype; therefore, it may be useful for selected patients who tolerate acidic foods, but inappropriate for patients with mucositis, reflux, oral pain, dental erosions, severe xerostomia or swallowing impairment. Product quality, regulatory status, dosing, timing before meals and reproducibility across cancer populations remain unresolved.

For immunotherapy-associated dysgeusia, miraculin is attractive for three reasons. First, it is directly aligned with the symptom mechanism: it modifies taste perception rather than claiming general anticancer activity. Second, it can be incorporated into dietitian-led food experimentation: for example, testing citrus, yogurt, fruit, chilled high-protein foods or other acidic vehicles shortly after intake. Third, it can be evaluated with patient-centered endpoints such as intake, appetite, body weight, quality of life and treatment persistence. However, safety and applicability must be individualized. Patients with mucositis, reflux, oral pain, dental erosions, swallowing problems, neutropenia or strict dietary restrictions may not be ideal candidates for acid-based flavor strategies. Product quality and regulatory status also matter.

Table 4 summarizes the available clinical evidence. Only two independent interventional cohorts were identified: the 8-patient crossover pilot reported by Wilken and Satiroff and the 31-patient CLINMIR pilot trial. The subsequent CLINMIR publications on oral microbiota, intestinal microbiota and inflammatory or cachexia-related biomarkers are secondary analyses of the same parent cohort rather than independent confirmatory studies. Accordingly, the number of publications should not be interpreted as multiple replications of clinical efficacy.

## 7. Broader Nutritional and Natural-Product Strategies

Natural taste modulation should not be reduced to a single product. Many patients benefit from food-first strategies: adjusting temperature, aroma, acid, umami, texture, moisture and aftertaste. Herbs, spices, citrus, vinegar, fermented flavors, broths and umami-rich foods may improve palatability for some patients, whereas others need bland, cold or low-odor foods [17]. Precision supportive care means testing rather than assuming. Broader nutritional strategies should be considered the clinical foundation, with natural taste modulators used only as adjuncts. Practical approaches include protein rescue plans using tolerated textures and temperatures, rotation of protein sources when meat becomes metallic or bitter, use of chilled or moist foods to reduce odor and aftertaste, individualized acid or umami testing, oral nutritional supplements selected according to sensory tolerance, and early dietitian follow up when weight loss or dietary narrowing appears. These strategies are not specific to miraculin and may be more broadly applicable across chemotherapy, radiotherapy and immunotherapy populations. Their evidence base is largely derived from oncology nutrition principles and symptom management practice rather than dysgeusia-specific randomized trials.

Polyphenol-rich plant foods, fermented foods and prebiotic fibers are also relevant, but the clinical claim should be nutritional and microbiome-supportive rather than anticancer. Diets rich in plant diversity and fiber may promote microbial diversity and short-chain fatty acid production, which may support immune homeostasis [30,31,32,39,43]. Nevertheless, severely immunocompromised patients may require food-safety adaptations, and high-fiber interventions can worsen bloating, diarrhea or early satiety in selected patients. Similarly, probiotics and live fermented products must be used carefully in hematologic malignancy patients because bloodstream infection, contamination and strain-specific effects are legitimate concerns. In many cases, dietitian-guided food patterns are safer than supplement-driven interventions. The relative clinical readiness, evidence strength, mechanistic clarity and research priority of these approaches are summarized in Table 5.

Clinical readiness reflects feasibility and compatibility with current supportive-care practice; evidence strength reflects the directness and quality of available clinical evidence for dysgeusia-related outcomes; mechanistic clarity reflects biological plausibility for improving taste perception, intake or oral–gut ecology. The table is intended to guide research prioritization and clinical caution rather than to rank anticancer efficacy.

## 8. Natural Products, Tumor Immunotherapy and Claim Discipline

Natural products and their derivatives have been extensively discussed as modulators of tumor immunity, but their role in supportive cancer care requires careful claim discipline. Deng and colleagues provided an important mechanistic framework by summarizing how natural products may influence immune cells, cytokines, chemokines, immune checkpoints, immunogenic cell death and intracellular signaling pathways involved in tumor immunity [23]. Subsequent reviews have further expanded this field by addressing tumor microenvironment remodeling, natural small molecules, regulatory immune cells, drug resistance and immunogenic cell death [24,25,26,27,28]. Examples include polyphenols, flavonoids, terpenoids, alkaloids and other plant-derived compounds that have been reported to influence inflammatory signaling, oxidative stress, immune-cell polarization, checkpoint pathways or immunogenic cell death in preclinical models. However, these mechanistic observations rarely translate directly into clinically validated immunotherapy adjuncts. Major barriers include low or variable bioavailability, differences between purified compounds and food matrices, batch-to-batch variability of phytochemical preparations, uncertain pharmacokinetics, potential interactions with anticancer drugs or anticoagulants, hepatotoxicity, contamination risk, immune effects that may be context-dependent and regulatory heterogeneity. For patients receiving immune checkpoint inhibitors, CAR T-cell therapy or bispecific antibodies, these uncertainties are clinically important because unmonitored supplements may interfere with safety assessment or supportive care attribution.

Building on this mechanistic foundation, the present manuscript defines a clinically cautious and translationally actionable boundary for patients receiving tumor immunotherapy. Natural products should first be evaluated for supportive-care outcomes such as symptom relief, nutritional adequacy, oral intake, quality of life, tolerability and treatment adherence. Potential antitumor immune modulation provides biological rationale for research, but it should not be communicated as clinical benefit unless supported by adequately designed intervention trials with predefined efficacy and safety endpoints. This distinction is essential to preserve scientific credibility, protect patient safety and guide a realistic pathway from biological plausibility to supportive care implementation.

## 9. Oral–Gut Microbiome Axis: Mechanistic Hypotheses for Future Trials

The oral–gut microbiome axis provides several testable hypotheses. This section is framed as mechanistic and hypothesis-generating. At present, oral-gut microbiome evidence is much stronger for immunotherapy response and treatment-related ecosystem disruption than for dysgeusia improvement. Therefore, microbiome endpoints should be embedded in future dysgeusia trials to generate mechanistic insight, but they should not yet guide routine clinical dysgeusia treatment decisions. First, oral dysbiosis may contribute to local inflammation, barrier dysfunction, altered saliva composition and taste receptor microenvironment. Second, dysgeusia-driven dietary narrowing may reduce substrate diversity for the gut microbiota, lowering resilience and short-chain fatty acid production. Third, antibiotics, antifungals, proton-pump inhibitors and hospitalization may disturb microbial communities at the same time that patients reduce intake. Fourth, changes in microbiome composition may interact with systemic immune tone, although the strongest clinical evidence currently comes from immune checkpoint inhibitor settings rather than bispecific antibodies [30,31,32,38,39,40,41,42,43].

A microbiome-informed dysgeusia trial should therefore combine clinical and biological endpoints. Clinical endpoints could include objective gustatory testing, patient-reported dysgeusia severity, appetite, dietary intake, weight trajectory, PG-SGA score, handgrip strength, EORTC quality-of-life domains, distress and treatment persistence. Biological endpoints could include oral swabs, saliva markers, stool microbiome, short-chain fatty acids, bile acid metabolites, inflammatory markers and medication exposures. Microbiome results should be interpreted as exploratory unless adequately powered and pre-specified.

**Figure 1:**
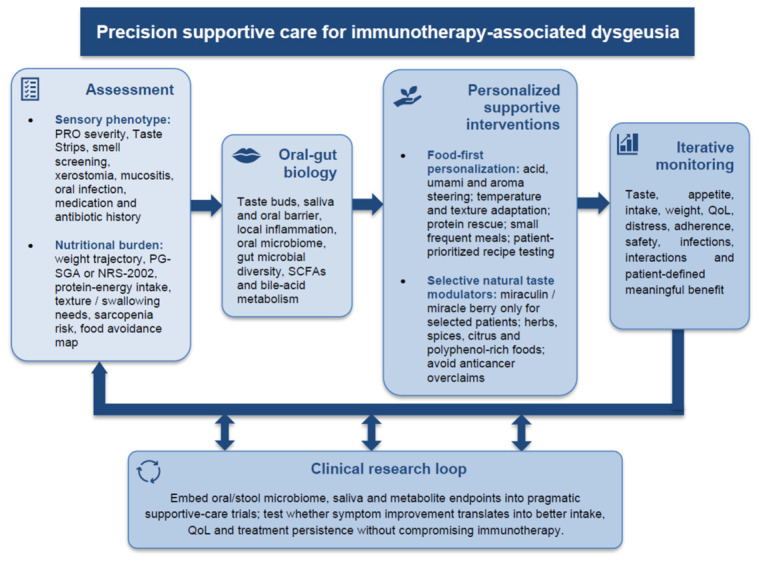
Precision supportive-care framework for dysgeusia-related nutritional burden during cancer immunotherapy. The model is intended for supportive care research and clinical decision support; it is not a substitute for oncology decision-making. Data capture occurs at four points: sensory phenotype assessment (patient-reported dysgeusia severity, Taste Strips, smell screening), nutritional monitoring (weight trajectory, protein-energy intake, PG-SGA or NRS-2002, sarcopenia risk), oral–gut biology (xerostomia, mucositis, oral infection, medication exposures, optional oral and stool microbiome diversity indices, short-chain fatty acids and inflammatory markers) and iterative outcome monitoring (PRO scores, QoL, distress, safety, treatment interruptions and patient-defined meaningful benefit). PRO, patient-reported outcome; QoL, quality of life; SCFAs, short-chain fatty acids.

Figure 1 summarizes the proposed framework. The model begins with sensory phenotyping because dysgeusia is not a uniform complaint. It then links the phenotype to nutritional risk, oral–gut biology, individualized food strategies, cautious natural taste modulation and iterative monitoring. The framework is cyclical: dysgeusia changes over time, immunotherapy regimens evolve, infections and antibiotics occur and patient priorities may shift. The goal is to preserve nutrition, autonomy, quality of life and therapeutic persistence.

## 10. Proposed Trial Design

The next step should be a staged supportive-care research program. Three designs are conceptually possible. First, a pragmatic bundled supportive-care trial could compare usual care with structured dysgeusia-focused supportive care, including taste testing, dietitian-led sensory phenotyping, individualized flavor and texture plans, protein rescue, oral-care optimization, weight and intake monitoring and brief psycho-oncological support for eating-related distress. This design would test real-world clinical effectiveness but would not allow attribution of benefit to a single component. Second, a miraculin-specific randomized trial could evaluate a standardized dried miracle berry or miraculin-containing product in predefined eligible patients with compatible sensory phenotypes and no contraindications to acidic food vehicles. This design would provide clearer efficacy attribution but would not address the broader nutritional and psychosocial burden. Third, a factorial or adaptive design could test a core supportive-care bundle with or without a standardized miraculin module, thereby separating the effect of structured nutritional care from the incremental value of natural taste modulation.

For high-burden immunotherapy populations, such as patients receiving GPRC5D-directed bispecific antibodies, a feasible first step would be a randomized pilot trial of usual care versus structured dysgeusia-focused supportive care, with an embedded optional or randomized miraculin module in eligible patients. The primary objective should be feasibility and signal detection rather than definitive efficacy. Key feasibility endpoints should include recruitment, retention, adherence to taste testing and food diaries, acceptability of the intervention, safety, missingness of patient-reported outcomes and feasibility of oral and stool sampling. A later adequately powered trial could use change in dysgeusia-related nutritional impact, protein energy intake or weight stability over 8 to 12 weeks as a primary endpoint.

Secondary endpoints should include objective taste function, patient-reported dysgeusia severity, appetite, PG-SGA score, EORTC QLQ-C30 appetite loss and global health status, eating-related distress, dietary diversity, treatment adherence, dose interruptions, patient satisfaction and safety. Exploratory endpoints could include oral and intestinal microbiome profiles, saliva markers, short-chain fatty acids, inflammatory biomarkers, antibiotic exposure and medication changes. Randomization should be stratified by baseline dysgeusia severity, xerostomia, treatment phase and recent antibiotic exposure. Blinding may be feasible for miraculin-specific trials using placebo products, but is unlikely for complex dietary-supportive-care bundles. Concomitant nutritional interventions should be documented prospectively to avoid misattribution of effect. A clinically meaningful improvement should be defined a priori, for example as maintenance of body weight within 2% of baseline, improvement in PG-SGA category, a prespecified increase in protein energy intake, or a prespecified improvement in patient-reported dysgeusia severity. The principal features, strengths and limitations of these trial designs are summarized in Table 6.

## 11. Clinical Implementation: What Can Be Done Now?

Before definitive intervention trials are available, several low-risk practices can be considered on the basis of established oncology nutrition principles, oral supportive care guidance and expert interpretation rather than dysgeusia-specific validated intervention trials. Patients starting high-risk immunotherapies should receive anticipatory counseling that taste changes may occur, that nutritional risk should be reported early and that supportive strategies can be tailored. Screening should include weight trajectory, appetite, oral symptoms, xerostomia, mucosal pain, dysphagia, food aversions, protein intake and psychosocial burden. This approach is consistent with guideline principles for early nutritional screening and symptom-oriented oncology supportive care [18,19,20,21,22].

For patients, the most helpful message is practical and validating: taste changes are real, they are not simply psychological and supportive strategies can be personalized. Eating may need to become experimental for a time, with structured testing of cold versus warm foods, acidic versus mild flavors, soft versus crunchy textures, liquid versus solid protein sources and different meal contexts. Psycho-oncological support can be valuable when food loses emotional meaning, family meals become stressful or the patient considers discontinuing treatment because eating has become intolerable. These measures should, therefore, be presented to patients as supportive, individualized and reversible strategies. They should not replace oncological toxicity management, formal nutritional assessment or oral medicine evaluation when clinically indicated.

## 12. Research Agenda

The most urgent research priorities are: first, longitudinal phenotyping of dysgeusia in immunotherapy populations, especially GPRC5D/talquetamab-treated patients; second, validation of clinically meaningful dysgeusia-related nutritional endpoints; third, feasibility testing of structured dietitian-led supportive care pathways; fourth, randomized evaluation of miraculin or other natural taste modulators in predefined sensory phenotypes; and fifth, exploratory integration of oral-gut microbiome, saliva, inflammatory and dietary diversity endpoints. Across these studies, patient-defined benefit should be included because meaningful improvement may consist not only of normalized taste, but also of maintaining protein intake, preventing further weight loss, sharing meals again or continuing effective immunotherapy with less distress.

## 13. Limitations

This review has limitations. It is not a systematic review and does not provide pooled estimates, formal risk-of-bias assessment or certainty grading. The available literature is heterogeneous and spans general cancer-related dysgeusia, chemotherapy, radiotherapy, head-and-neck cancer, malnourished oncology populations, immune checkpoint inhibitor studies, CAR T-cell therapy, bispecific antibodies and microbiome immunotherapy research. These populations differ substantially in treatment mechanisms, oral toxicity patterns, nutritional vulnerability and expected duration of symptoms, which limits direct comparability.

Evidence for immunotherapy-associated dysgeusia is still uneven, with the strongest direct signal currently coming from GPRC5D-directed therapy in multiple myeloma. Evidence for miraculin and dried miracle berry products in cancer dysgeusia is promising but based on small studies in selected populations; direct evidence in immunotherapy-associated dysgeusia is not yet established. The possibility of publication bias toward positive pilot studies should be considered. Microbiome associations with immunotherapy response are biologically compelling but heterogeneous across cancer types, cohorts, sequencing methods, dietary exposures and antibiotic use, and they do not prove that microbiome modulation improves dysgeusia. Therefore, the proposed framework should be understood as a research guiding and clinical supportive model rather than a validated treatment algorithm or clinical standard.

## 14. Conclusions

Immunotherapy-associated dysgeusia is an under-standardized nutrition impact toxicity with consequences for appetite, dietary diversity, weight, quality of life and treatment persistence. Talquetamab and other GPRC5D-directed therapies highlight the need for supportive care models that integrate sensory phenotyping, nutritional assessment, oral health evaluation, patient-centered outcomes and exploratory oral–gut microbiome biology. Natural taste modulators, especially miraculin-rich miracle berry products, provide a plausible and innovative research candidate for selected patients, but current evidence remains preliminary and direct efficacy in immunotherapy-associated dysgeusia has not been established. The central research question is not whether natural products can be broadly promoted as anticancer adjuncts, but whether carefully selected taste modulating and nutrition-supportive interventions can help patients maintain intake, quality of life and treatment persistence without compromising immunotherapy safety or efficacy. The framework proposed here should therefore be regarded as a translational research roadmap for precision supportive cancer care rather than a validated clinical standard.

## Figures and Tables

**Table 1:** Evidence context and approximate burden of dysgeusia across cancer treatment settings.

Treatment Setting	Typical Evidence Base	Reported Dysgeusia/Taste Alteration Burden	Interpretation for This Review
Chemotherapy	Systematic reviews, meta-analyses and observational studies across heterogeneous tumor types and regimens	Common, but estimates vary substantially by agent, assessment method and treatment phase	Strong background evidence, but mechanisms and management may not directly translate to immunotherapy
Radiotherapy/ head-and-neck cancer	Prospective cohorts, symptom studies and supportive care literature	Often high when the oral cavity, salivary glands or taste structures are exposed	Highly relevant for pathophysiology and supportive care, but anatomically and mechanistically distinct from systemic immunotherapy
Immune checkpoint inhibitors	Mostly indirect evidence from oral toxicity, nutrition and microbiome literature	Less consistently reported as a discrete endpoint	Evidence remains indirect; microbiome data mainly concern treatment response rather than dysgeusia
CAR T-cell therapy	Sparse dysgeusia-specific reporting; broader supportive-care and oral-complication literature	Not yet well quantified as an independent supportive care endpoint	Important comparator immunotherapy setting, but current evidence is limited
GPRC5D/ talquetamab- directed therapy	Clinical trials, supportive-care reports and emerging patient-centered studies	Frequently reported, but estimates are not directly comparable because trial adverse-event groupings, retrospective CTCAE grading and taste-focused patient-reported or psychophysical assessments measure different constructs	Most direct immunotherapy-associated model for dysgeusia-focused supportive-care research

**Table 2:** Study-level evidence on talquetamab-associated taste disturbance and related oral outcomes.

Study	Design and Population	n and Dosing/Exposure	Follow-Up or Assessment Timing	Assessment and Definition	Taste Outcome and Interpretive Caveat
Chari et al., 2022 [9]	Phase 1 MonumenTAL-1; heavily pretreated relapsed/refractory multiple myeloma	30 at 0.405 mg/kg weekly; 44 at 0.8 mg/kg every other week	Median 11.7 and 4.2 months, respectively	Investigator-reported treatment-emergent adverse events; the published category ‘dysgeusia’ did not provide psychophysical taste testing	63% and 57%, respectively; trial adverse event incidence, not a phenotype-specific prevalence estimate
Naqvi et al., 2024 [12]	Single-center retrospective cohort	17 patients receiving at least one talquetamab dose	Treatment through December 2022; duration varied; persistence assessed after discontinuation where available	CTCAE-graded dysgeusia (grade 1 or 2) and serial weight data	14/17 (82%); small retrospective cohort, with two patients reporting persistence beyond 4 months after discontinuation
Fleischer et al., 2025 [13]	Prospective multicenter observational comparison	26 talquetamab-treated patients; assessed after at least two doses; comparator groups: melphalan/ASCT n = 35 and BCMA bispecifics n = 26	Cross-sectional assessment during treatment	Taste Strips, olfactory testing and questionnaires; patient-reported marked taste decline was distinct from objective test performance	25/26 (96.2%) reported marked decline; taste-focused assessment and construct differ from routine adverse event reporting
Fleischer et al., 2026 [17]	Prospective exploratory mixed-methods patient-experience cohort	25 talquetamab-treated patients selected for new-onset taste change and at least 3 months of exposure	Single structured assessment during ongoing treatment	Patient-reported taste change and xerostomia symptoms; no psychophysical taste test or sialometry	Not suitable for prevalence estimation because taste change was an inclusion criterion; informs phenotype, food tolerance and supportive-care needs

**Table 3:** Dysgeusia-oriented supportive care assessment for patients receiving cancer immunotherapy. The table integrates guideline-consistent nutritional and oral care principles with dysgeusia-specific expert interpretation.

Domain	Recommended Assessment	Clinical Purpose	Evidence Basis
**Taste phenotype**	Patient-reported dysgeusia severity; Taste Strips or other validated gustatory testing; affected taste qualities; onset and course.	Separates global complaint from measurable sensory pattern; allows monitoring.	Validated tool/direct assessment
**Flavor and smell**	Brief smell history or validated olfactory screening when available; retronasal flavor complaints.	Distinguishes taste loss from flavor loss and prevents misdirected interventions.	Validated tool/clinical extrapolation
**Oral compartment**	Xerostomia, mucositis, candidiasis, dental disease, tongue changes, oral pain, swallowing symptoms.	Identifies treatable contributors and safety risks.	Guideline-consistent clinical assessment
**Nutrition**	Weight trajectory; food diary; protein-energy intake; PG-SGA or NRS-2002; dietary diversity; texture limitations.	Detects clinically relevant nutrition-impact burden early.	Validated tools/guideline-consistent
**Treatment context**	Immunotherapy type; treatment phase; corticosteroids; antibiotics; antifungals; proton-pump inhibitors; comorbidities.	Frames dysgeusia within interacting medical and microbiome exposures.	Expert interpretation/exposure assessment
**Psychosocial impact**	Food-related distress; avoidance of social eating; fear of treatment discontinuation; patient-defined goals.	Captures quality-of-life burden and adherence threat.	Expert interpretation/supportive-care assessment

**Table 4:** Clinical evidence on miracle berry- and miraculin-based interventions in patients with cancer-related taste disorders.

Study	Design	Cancer Type/Setting	n	Intervention	Comparator	Outcomes	Main Finding	Evidence Level
Wilken and Satiroff, 2012 [52]	Randomized, placebo-controlled two-period crossover pilot; 4 weeks	Heterogeneous cancers; chemotherapy-associated taste changes	8	Miracle fruit supplement for 2 weeks	Placebo for 2 weeks; crossover sequence	Daily food/beverage intake; patient-reported taste change and palatability	All participants reported positive taste changes during miracle fruit exposure; outcomes were subjective and the sample was extremely small	Very low-certainty direct clinical evidence
López-Plaza et al., 2024; CLINMIR primary study [53]	Exploratory randomized, parallel-group, triple-blind, placebo-controlled pilot; 3 months	Heterogeneous cancers; active antineoplastic treatment; malnutrition and taste disorders	31 randomized; 21 completed	Standard dose: 150 mg dried miracle berry plus 150 mg freeze-dried strawberry; high dose: 300 mg dried miracle berry before each main meal	300 mg freeze-dried strawberry before each main meal	Electrogustometry; chemical taste perception; dietary intake; nutritional and morphofunctional status; quality of life; safety	Standard dose improved electrogustometric taste acuity over time and salty-taste scores versus placebo; signals favored energy coverage, fat-free mass and selected quality-of-life outcomes; no intervention-related adverse events	Low-certainty preliminary randomized evidence
Plaza-Diaz et al., 2024; oral microbiome analysis [54]	Exploratory secondary analysis of the CLINMIR cohort	Same parent cohort	31 randomized	Same standard- and high-dose regimens	Placebo	Salivary microbiome diversity and taxonomic composition; associations with taste, diet, mucositis and cytokines	Selected taxonomic changes were observed, including lower abundance of Veillonella and specific Streptococcus/Veillonella species in the standard-dose group; no causal mediation of taste improvement was shown	Very low-certainty exploratory mechanistic evidence
Plaza-Diaz et al., 2025; intestinal microbiome analysis [55]	Exploratory secondary analysis of the CLINMIR cohort	Same parent cohort	31 randomized; 21 completed	Same standard- and high-dose regimens	Placebo	Stool microbiome composition and alpha diversity; plasma SCFAs; correlations with diet, taste and inflammatory markers	No treatment-by-time effect for overall alpha-diversity; selected taxonomic changes and increased plasma acetate were exploratory	Very low-certainty exploratory mechanistic evidence
Álvarez-Mercado et al., 2025; biomarker analysis [56]	Exploratory secondary biomarker analysis of the CLINMIR cohort	Same parent cohort	31 enrolled in parent trial	Same standard- and high-dose regimens	Placebo	Inflammatory and cachexia-related biomarkers; associations with sensory and nutritional variables	IFN-gamma decreased in the standard-dose group, but several changes were nonspecific and no significant associations with sensory or nutritional outcomes were demonstrated	Very low-certainty exploratory biomarker evidence

Abbreviations: DMB, dried miracle berry; IFN-gamma, interferon gamma; SCFAs, short-chain fatty acids. Evidence levels represent a narrative appraisal of directness, design robustness, sample size, attrition, outcome validity and multiplicity of analyses; they do not constitute a formal GRADE assessment. The CLINMIR primary, oral microbiome, intestinal microbiome and biomarker publications originate from the same randomized pilot cohort and should not be interpreted as independent replications. None of the available studies specifically evaluated immunotherapy-associated or GPRC5D/talquetamab-associated dysgeusia.

**Table 5:** Evidence-readiness map for supportive interventions in immunotherapy-associated dysgeusia.

Intervention	Clinical Readiness	Evidence Strength	Mechanistic Clarity	Priority	Key Limitation
Systematic taste phenotyping	High	Moderate	High	High	Requires workflow integration and staff training
Dietitian-led flavor and texture steering	High	Moderate	Moderate	High	Individual response varies; dysgeusia-specific trial data remain limited
Protein rescue plan	High	High for oncology nutrition; indirect for dysgeusia	Moderate	High	Intake may remain limited if taste aversion is severe
Oral care optimization	High	High for oral complications; indirect for dysgeusia	High	High	Requires access to dental/oral medicine expertise in complex cases
Miraculin/dried miracle berry	Moderate	Low; pilot data only	High for taste modulation	Trial-preferred	No direct evidence in immunotherapy-associated dysgeusia; product quality and eligibility matter
Plant-forward, fiber-rich dietary pattern	Moderate	Moderate for microbiome/immunotherapy response; indirect for dysgeusia	Moderate	Medium	Adapt for neutropenia, gastrointestinal symptoms and intake limitations
Probiotics or live fermented products	Low-to-moderate	Low and strain-specific	Moderate	Low outside trials	Infection risk and uncertain immunotherapy interactions in vulnerable patients
High-dose supplements/herbal extracts	Low	Low for dysgeusia; mostly preclinical for immunomodulation	Variable	Avoid routine use	Interactions, contamination, hepatotoxicity and overclaiming risks

**Table 6:** Trial-ready design options for precision supportive care in immunotherapy-associated dysgeusia.

Design Element	Pragmatic Bundle Trial	Miraculin-Specific Trial	Factorial/Adaptive Design
Main question	Does structured dysgeusia-focused supportive care improve nutritional and patient-centered outcomes compared with usual care?	Does a standardized miraculin-containing product improve taste-related outcomes in eligible patients?	What are the independent and incremental contributions of the supportive care bundle and miraculin?
Population	Adults receiving high-burden immunotherapy with moderate-to-severe dysgeusia	Patients with dysgeusia who tolerate acidic food vehicles and have no relevant contraindications	Same as bundle trial, with predefined eligibility for intervention modules
Comparator	Usual care	Placebo or matched control product	Usual care and/or factorial component allocation
Primary early endpoint	Feasibility, acceptability, adherence and safety	Change in taste perception or dysgeusia-related food acceptability	Feasibility plus component-specific signal detection
Potential efficacy endpoint	Protein energy intake, weight stability or dysgeusia-related nutritional impact at 8–12 weeks	Patient-reported taste improvement, objective taste testing and food acceptability	Same endpoints with component-level attribution
Secondary endpoints	PG-SGA, EORTC QLQ-C30, appetite, distress, dietary diversity and treatment persistence	Appetite, intake, PG-SGA, quality of life and safety	Same as bundle trial
Exploratory endpoints	Oral and stool microbiome, saliva, SCFAs, inflammatory markers and antibiotic exposure	Optional oral microbiome and biomarker endpoints	Embedded translational endpoints
Strength	Real-world supportive care relevance	Clearer attribution to miraculin	Separates bundle and modulator effects
Limitation	Cannot attribute benefit to one component	Narrower clinical scope	More complex design and larger sample size requirements

## Data Availability

No new data were generated or analyzed in this review article. Data sharing is not applicable.

## Supplementary Materials

The following supporting information can be downloaded at: https://www.mdpi.com/article/10.3390/nu18142393/s1, Supplementary Table S1. Focal PubMed search strings used for the structured evidence map (last update: 16 July 2026).
