# Supplementary material for: Natural Taste Modulators and Microbiome-Aware Nutritional Support for Immunotherapy-Associated Dysgeusia: A Translational Perspective for Precision Supportive Cancer Care
Source: Nutrients. 2026 Jul 22;18(14):2393. doi: 10.3390/nu18142393 (PMC13415068; doi:10.3390/nu18142393)
Supplement: Supplementary file 1 [file nutrients-18-02393-s001.zip › nutrients-4412282-supplementary.pdf]

Supplementary Table S1. Focal PubMed search strings used for the structured evidence map (last update: 16 July 2026).

Stream	PubMed search string	Records
Cancer-related dysgeusia	(dysgeusia[Title] OR "taste alteration"[Title] OR "taste disorder"[Title]) AND (cancer[Title/Abstract] OR oncology[Title/Abstract])	92
Talquetamab/GPRC5D	(talquetamab[Title/Abstract] OR GPRC5D[Title/Abstract]) AND (dysgeusia[Title/Abstract] OR taste[Title/Abstract] OR xerostomia[Title/Abstract] OR "oral toxicity"[Title/Abstract] OR "weight loss"[Title/Abstract])	33
Miraculin/miracle berry	(miraculin[Title/Abstract] OR "miracle berry"[Title/Abstract] OR "miracle fruit"[Title/Abstract] OR "Synsepalum dulcificum"[Title/Abstract]) AND (cancer[Title/Abstract] OR dysgeusia[Title/Abstract] OR "taste disorder"[Title/Abstract] OR malnutrition[Title/Abstract])	19
Microbiome and immunotherapy	("gut microbiome"[Title] OR "oral microbiome"[Title]) AND (immunotherapy[Title/Abstract] OR "immune checkpoint"[Title/Abstract]) AND (diet[Title/Abstract] OR nutrition[Title/Abstract] OR antibiotic*[Title/Abstract] OR response[Title/Abstract])	219
